# Efficacy of increasing levels of exposure therapy in the treatment of maladaptive behaviors and anxiety

**DOI:** 10.3892/mi.2023.115

**Published:** 2023-10-03

**Authors:** Fatih Bal

**Affiliations:** Department of Psychology, Faculty of Humanities and Social Sciences, Sakarya University, Serdivan, Sakarya 54187, Turkey

**Keywords:** cortical function, electroencephalography, treatment, mental disorder, anxiety

## Abstract

The present study examined the effectiveness of increasing levels of exposure therapy, which is applied for the treatment of maladaptive behaviors and anxiety. A total of 16 sessions were applied to the study group in the experimental group three times a week for 10 weeks. Patients aged ≥18 years whom the referring clinician evaluated as meeting the criteria for the Diagnostic and Statistical Manual of Mental Disorders, fourth edition (DSM-V-TR) Generalized Anxiety Disorder were included in the study. For the control group, demographic characteristics and Spielberger's State-Trait Anxiety Inventory were applied in the first session, followed by Spielberger's State-Trait Anxiety Inventory as a post-test and follow-up. Electroencephalography (EEG) recordings of the study group were obtained at the cortical level. Electrodes for EEG measurements were recorded using the International 10/20 Electrode Placement System. EEG data were obtained using the EEG Analysis Program software. Following the data collection phase, all data were entered into cells based on items using SPSS 25 software. When the findings obtained in the study were examined, it was determined that the increasing levels of exposure and behavioral therapy applied for maladaptive anxiety decreased the anxiety levels compared to those before therapy. This finding can be interpreted as that the cortical function-oriented application method for anxiety effectively reduced the anxiety levels of the study group. However, EEG asymmetry revealed a change in the data before and after the application. These findings demonstrate that the application affects the EEG asymmetry changes at the cortical level.

## Introduction

The expansion of the prefrontal cortex (PFC) in the human brain is considered to be responsible for the subjective experience of anxiety and the ability to regulate anxiety-related responses (1,2). While these abilities are advantageous for adapting to the environment, they also contribute to developing anxiety disorders (3). The PFC gathers information from various cortical and subcortical structures to plan adaptation actions. Understanding the function of the PFC has been aided by organizing principles that describe function gradients across the PFC, such as the configuration of the lateral PFC (LPFC) along the rostral/caudal axis based on the level of cognitive control (4). Research using neuroimaging and lesion analysis has suggested that rostral areas of the LPFC are involved in higher levels of cognitive control, processing more abstract representations (5).

The hippocampus, known for its role in memory formation, is also crucial in regulating emotions during fear conditioning (6). Unlike the amygdala, which processes cues anticipating a threat, the dorsal hippocampus provides contextual information about the specific threat cue through interactions with the amygdala and ventromedial PFC (7). The dorsal hippocampus encodes contextual information about the threat or cues learned when presented with a stimulus, allowing the organism to differentiate between threat and safety cues, and to evaluate the threat level associated with the stimulus (8,9). This ability to distinguish between threatening and safe features may be essential for automatic cognitive appraisal and reappraisal, contributing to emotion regulation. In animal studies, mice exposed to a tone predicting a shock learn to fear both the tone and the context in which the tone-shock pairs occur (10). Understanding the involvement of the hippocampus in emotional regulation can provide insight into the mechanisms through which emotions are processed and controlled.

Understanding the role of the PFC in anxiety disorders has been challenging due to the complex nature of neural processes associated with anxiety. The PFC is responsible for assessing the likelihood of a threat in the environment by gathering information from different brain regions (11). Distorted threat estimations can create a cycle of overreactions to perceived dangers, leading to anxiety and avoidance behaviors. The frontal lobe, specifically the PFC, has significantly evolved in primates, and abnormalities in prefrontal function have been linked to anxiety disorders and their symptoms (2). Therefore, the present study aimed to determine the effectiveness of increasing levels of exposure therapy, in reducing anxiety levels and combating maladaptive behaviors. Participants were first assessed according to Spielberger's State-Trait Anxiety Inventory and were included in the study according to whether they were anxious (score >17). Participants were randomly divided into the experimental and control groups, with a total of 30 participants.

## Subjects and methods

### Study design and ethics approval

The author initiated the data collection process after obtaining permission from the Ethics Committee of Sakarya University (registration no. 22.11.1122); written informed consent was obtained from all subjects. Subjects were under no obligation to participate in the study and were assured of the confidentiality of all information.

The present study was an experimental research study based on cause-and-effect relationships, using a pre-test and post-test control group design. Experimental studies examine the change caused by one or more dependent variables on an independent variable, providing accurate results using comparable methods. However, the present study was designed as a quasi-experimental model. The pre-test and post-test control group design is commonly employed in comparison studies conducted using experimental models. The present study specifically employed the pre-test post-test control group design (https://quantifyinghealth.com).

Within the scope of the research, two groups, one experimental and one control, were formed. Prior to the application, all participants in the experimental and control groups were tested for their anxiety levels. In order to strengthen the internal validity of the study, a pre-test comparison was made between the experimental and control groups. In the present study, the therapeutic application was applied to the participants in the experimental group for a total 36 sessions, three times a week for 10 weeks. No intervention was applied to the participants in the control group.

### Participants

**Figure 1:**
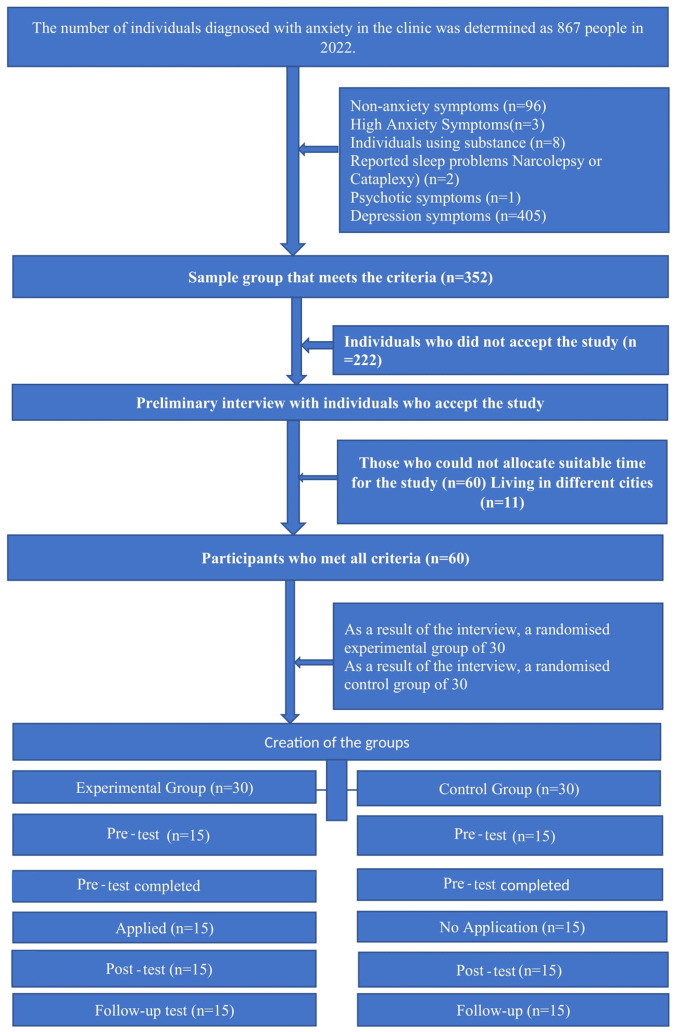
Flow chart of inclusion protocol for participants in the present study.

The study group comprised 60 individuals with anxiety disorders who applied to a private health clinic in Istanbul, Turkey. The selection of the working group and the flow chart are presented in Fig. 1.

The inclusion and exclusion criteria for the study were as follows: Participants were recruited from the clinical population and were reviewed separately by the referring psychiatrist. However, patients who ≥18 years of age and judged by the referring clinician to meet the criteria for Generalized Anxiety Disorder in the Diagnostic and Statistical Manual of Mental Disorders, Fourth Edition (DSM-V-TR) (12) were included. Participants were excluded if they expressed acute suicidal or homicidal ideation or showed acute psychotic symptoms. In addition, the exclusion criteria were as follows: The primary or secondary diagnosis met the criteria for obsessive-compulsive disorder, post-traumatic stress disorder, panic disorder, agoraphobia or acute stress disorder, the primary diagnosis was not an anxiety disorder, mental retardation, pervasive developmental disorder, psychosis, oppositional defiant disorder, conduct disorder or substance use was a co- or secondary diagnosis, the presence of a chronic disorder of organic origin and a recent trauma with ongoing forensic process.

Prior to commencing the research, all necessary units of the private health institution were informed about the research and the process, and the necessary permissions were obtained. All persons, units and institutions participating in the process were informed, and their application and research permissions were obtained.

### Study procedure

At this stage, the participants in the study group were informed about the increasing levels of exposure therapy and cognitive behavioral therapy.

### Sessions

After meeting with the client, a detailed conversation about anxiety, the treatment process for the client, and short and long-term treatment processes were determined. A form was prepared by discussing anxiety-related issues with each client and creating a list. Brain-based explanations for anxiety were given to each client. Short psychoeducational presentations were made for questions, such as how the brain works and what it needs. After the presentations, a form was created that included a list of clients who would participate in the exposure therapy and cognitive behavioral therapy. The goals selected from the list were divided into achievable tasks to gradually relieve the clients' anxiety. Behavioral changes were made by changing each client's behaviors towards different goals in this way and dividing them into small, easy practices. Clients were exposed to situations that aroused the sensation that they had difficulty in doing with increasing frequency, and the brain was reprogrammed. Any tasks that the clients could not carry out during the session were given to them as a home assignment. Unfulfilled home assignments were requested again, and support was provided for carrying out the home assignments. In this manner, the clients were provided with their comfort zones with measured and frequent steps, and the control of their home assignments was ensured. It was explained to the clients that moderate and reasonable anxiety strengthens memory. Thus, increasing levels of adrenaline in the brain play a role in encoding and reinforcing the implicit memory of the amygdala. This information enabled the clients to perform their home assignments meticulously. At the end of the application, the clients were informed that the research results would be shared and were thanked for their participation.

### Assessment measurements

Anxiety levels for the experimental group were measured using Spielberger's State-Trait Anxiety Inventory (pre-test and post-test) immediately before and after using the therapeutic method. After applying this therapeutic method, the anxiety levels were measured again. The demographic characteristics of the participants and Spielberger's State-Trait Anxiety Inventory were filled in the first session for the control group; the Spielberger's State-Trait Anxiety Inventory was then completed again. The measurement tools used are described below.

### Personal information form

This form included eight questions about sex, economic status, employment status, educational status and personal health.

### Spielberger's State-Trait Anxiety Inventory

The scale measures normal and abnormal traits and state anxiety levels of individuals (3). There are a total of 40 short statements on the scale. The first 20 items measure the level of anxiety related to the situation, and each item is graded as ‘Not at all’ (1 point), ‘Somewhat’ (2 points), ‘Very’ (3 points) and ‘Totally’ (4 points). A number of items were reverse scored (items 1, 2, 5, 8, 10, 11, 15, 16, 19 and 20). State anxiety scores are obtained by adding the constant v0, the constant value of the state anxiety scale, to the value obtained by subtracting the total score of the reverse-coded items from the total score of the directly coded items. Items 21 to 40 of the scale measure the trait anxiety level of the individual, and each statement is scored as ‘Not at all’ (1 point), ‘A little’ (2 points), ‘Very’ (3 points) and ‘Totally’ (4 points). In this section, seven items are reverse-coded (articles 21, 26, 27, 33, 36 and 39 contain these items). The trait anxiety level of the individual is obtained by subtracting the total score of the reverse-coded items from the total score of the directly coded items and adding 35, which is the constant value of the trait anxiety scale. It states that 0-19 points obtained from the scale do not indicate anxiety, 20-39 points indicate mild anxiety, 40-59 points indicate moderate anxiety, and 60-79 points indicate severe anxiety; individuals with a score ≥60 required professional assistance (4).

### Electroencephalography (EEG) measurements

Electrodes for EEG measurements were recorded using the International 10/20 Electrode Placement System. EEG data were visually scored for artifacts from blinking, eye movements and other motor movements when amplitudes exceeded ±50 V using the EEG Analysis Program software (WinEEG 256 EEG channels for QEEG/ERP processing and analysis) All artifact-free EEG data were analyzed using a 1-second-wide discrete Fourier transform (DFT). Power (square microvolts) was derived from the DFT output in three alpha frequency bands (alpha-I: 8-10 Hz; alpha-II: 10-13 Hz; full alpha: 8-13 Hz).

### Statistical analyses

After the data collection phase was over, all data were entered into the cells on an item basis using the Statistical Program for Social Sciences 25 00 package program (IBM Corp.), and the total scores obtained from the scales were obtained.

The data obtained from the personal information form were analyzed using the descriptive analysis method, and the frequency and percentage distributions of the sociodemographic information of the study group were obtained. It was examined whether the data obtained exhibited a significant difference before and after the therapeutic application in the experimental and control groups. The data collected in the experimental and control groups were analyzed using descriptive and inferential statistical tests, such as an independent t-test (to compare the average depression level in the two groups) and a paired t-test (to compare the average depression level) using the SPSS-25 program to determine the anxiety level before and after the intervention. Moreover, the Chi-squared test was used to investigate the demographic characteristics of the study participants. Participants' Spielberger's Trait-State Anxiety Inventory, pretest, posttest, and follow-up test scores were analyzed by ANOVA and ANCOVA and multiple comparisons were analyzed using Tukey's HSD test. Finally, reliability analyses were conducted for the reliability of the scale used in the study. The results were evaluated at 95% confidence intervals. A value of P<0.05 was considered to indicate a statistically significant difference.

## Results

### Demographic characteristics of the study participants

All the demographic characteristics of the study participants are presented in Table I. The Chi-squared test was used to analyze demographic variables to determine whether the participants in the study were equally distributed in the experimental and control groups. Considering the sex variable of the experimental group in the study, the number of males was 46.7% (n=7) and that of females was 53.3% (n=8). The sex variable in the control group was considered; the number of males was 53.3.7% (n=8) and that of females was 46.7% (n=7). Considering the age variable of the experimental group, the number of participants between the ages of 18-25 years was 40.0% (n=6), that between 26-45 years was 20.0% (n=3), and the number of participants ≥46 years of age was 40.0% (n=6). Considering the age variable of the control group, the number of participants between the ages of 18-25 years was 40.0% (n=6) and the number of participants between the ages of 26-45 years was 20.0% (n=3). The number of participants over the age of 46 was 40% (n=6). Considering the educational variable of the experimental group, the number of primary school graduates was 40.0% (n=6), the number of secondary school graduates was 20.0% (n=3), the number of high school graduates was 13.3% (n=3), and that of university graduates was 26.7% (n=4). When the education variable of the control group was considered, the number of primary school graduates was 40.0% (n=6), the number of secondary school graduates was 20.0% (n=3), the number of high school graduates was 13% (n=3), and the number of university graduates was 26.7% (n=4). Considering the drug use variable in the experimental group, the number of participants using drugs was 40.0% (n=6), and the number of participants not using drugs was 60.0% (n=9). The number of participants using drugs (anti-depressants, benzodiazepine or others) was 53.3% (n=9) when looking at the variable of drug use in the control group. =8), and the number of participants who did not use drugs was 46.7% (n=9). Considering the marital status variable of the experimental group, the number of single participants was 46.7% (n=7), and the number of married participants was 53.3% (n=8). When the marital status variable of the control group was examined, the number of single participants was 40.0% (n=6), and the number of married participants was 60.0% (n=9) (Table I). As shown by these demographic variables, there were no statistically significant differences in demographic characteristics such as sex, education level, age, and drug use between the two groups and the characteristics were homogeneous (P>0.05).

### Pre-test, post-test and follow-up test scores

As shown in Table II, in the experimental group, there was a significant difference between the participants' Spielberger's State-Trait Anxiety Inventory pre-test, post-test and follow-up test scores (Wilk's λ, 800=F (1.14)=29.122, P<0.001 ɳ2=0.530) and the effect size was calculated to be high. The post-test mean score (x̄=4.6667) and follow-up test mean score (x̄=4.4667) were lower than the pre-test mean score(x̄=8.0667) in Table III. On the other hand, in the control group the difference between the post-test and follow-up test scores was not significant. This finding shows that the participants' anxiety levels significantly decreased after the application and in the measurements made after the application, and the results did not change in the follow-up measurements made afterward. This finding shows that the effect of the application made in the research continues. The descriptive analysis results of the pre-test, post-test and follow-up test scores of the experimental and control groups are presented in Table III.

**Figure 2:**
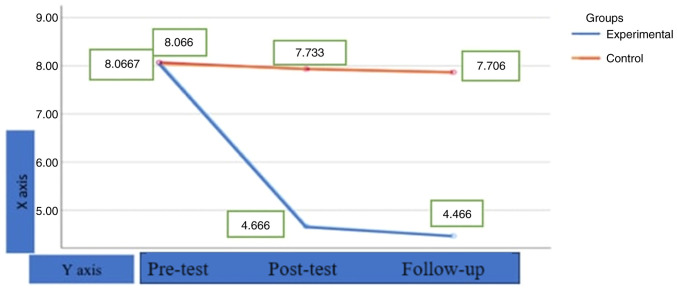
Results of repeated measurements analysis of the pre-test, post-test and follow-up test scores of the study participants.

As shown in Fig. 2, the pre-test Trait-State Anxiety mean score of the experimental group was 8.0667, the post-test mean score was 4.6667, and the follow-up mean score was 4.4667. The pre-test Trait-State Anxiety mean score of the control group was 8.0667, the post-test mean score was 7.733, and the follow-up mean score was 7.706. This finding indicates that the participants' anxiety levels markedly decreased after the application and in the measurements made after the application, and the results did not change in the follow-up measurements made afterwards. This finding indicates that the effect of the therapeutic application made in the research continues. On the other hand, it can be seen that there is no change in the control group. As shown in Fig. 2, it is clear that the change is due to the therapeutic application.

In addition, preliminary checks were made to confirm that the variances were not violated, reliable measurement of normality, linearity, homogeneity of variances, and homogeneity of regression trends. According to the results of ANCOVA, no significant difference was found between the post-test mean scores adjusted for Spielberger's State-Trait Anxiety Inventory according to sex (P>0.05) (Table IV). In other words, the results indicate that females and males do not react differently to the therapeutic approach used in the study.

### EEG asymmetry changes at the cortical level

**Figure 3:**
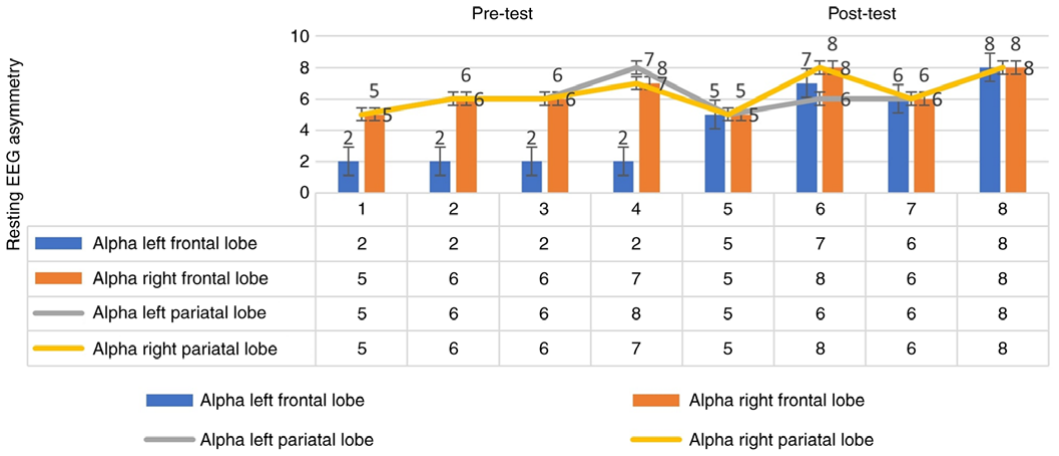
Pre-test and post-test resting EEG analysis results of the participants in the experimental group. EEG, electroencephalography.

As shown in Fig. 3, in the pre-test, in the first measurement, the pre-test alpha frequency of the right frontal lobe was 5 Hz and that of the left frontal lobe was 2 Hz; in the second measurement, the alpha frequency of the right frontal lobe was 6 Hz and that of the left frontal lobe was 2 Hz; in the third measurement, the alpha frequency of the right frontal lobe was 6 Hz and that of the left frontal lobe was 2 Hz; in the fourth measurement, the alpha frequency of the right frontal lobe was 7 Hz and that of the left frontal lobe was 2 Hz. In the post-test, in the fifth measurement, the alpha frequency of the right frontal lobe was 5 Hz and that of the left frontal lobe was 5 Hz; in the sixth measurement, the alpha frequency of the right frontal lobe was 8 Hz and that of the left frontal lobe was 7 Hz; in the 7th measurement, the alpha frequency of the right frontal lobe was 6 Hz and that of the left frontal lobe was 6 Hz; in the 8th measurement, the alpha frequency of the right frontal lobe was 8 Hz and that of the left frontal lobe was 8 Hz. As can be seen by the graph in Fig. 3, a change was observed in the pre-test. There was no change in the post-test. These findings indicate that there was a marked interaction of the application according to the frontal region. Therefore, this indicates that the application affects EEG asymmetry changes at the cortical level.

The changes in the frontal and parietal lobe frequencies were examined. In the experimental group, the analysis was performed separately for the resting EEG asymmetry alpha right and left before and after the therapeutic application. It was determined that there was a significant interaction at 10-13 Hz for alpha right and left relative to the frontal region [t (14)=1.344, P<0.05]. This indicates a change in EEG asymmetry (please also see the aforementioned data; Fig. 3). In the experimental group, the analysis was performed separately for the resting EEG asymmetry alpha right and left before and after the application. It was determined that there was a significant interaction to the parietal region at 10-13 Hz for alpha right and left [(t (14)=3.111, P<0.05] (Table V) This indicates a change in EEG asymmetry asymmetry (please also see the aforementioned data; Fig. 3). These findings demonstrated that the therapeutic application affected the EEG asymmetry changes at the cortical level.

## Discussion

In the present study, when the data obtained from the analyses were examined, the effectiveness of the gradually increasing levels of exposure therapy and cognitive behavioral therapy for maladaptive behavior and anxiety was examined. To date, to the best of our knowledge, there are no studies available in Turkey on the application of such methods, namely a brain-based psychological intervention program for anxiety. Therefore, the study is the first of its kind in Turkey. Herein, it was found that the methods used for maladaptive behavior and anxiety were effective and reduced the levels of anxiety in the study participants. This finding can be interpreted as an effective application method for anxiety.

According to the follow-up evaluations at the end of the therapy, it was stated by all clients that they benefited greatly from the therapeutic application and that their quality of life increased compared to that before the therapy. A previous study on cognitive behavioral therapy demonstrated that the participants experienced a significant shift in their resting forebrain activity from greater relative right to greater relative left after receiving treatment (13). That study also found that individuals with higher levels of left frontal brain activity before treatment had larger reductions in anxiety symptoms after treatment and lower levels of anxiety post-treatment (11). These associations were specifically related to the frontal alpha EEG asymmetry metric. These findings suggest that resting frontal EEG asymmetry may indicate symptom improvement and overall functioning in anxious patients who receive effective psychological treatment (11). The results of the present study are supported by other international research that has shown alterations in neural circuits related to anxiety disorders, such as exaggerated amygdala responses and impaired regulation by the PFC and hippocampus (14,15). Exposure to chronic stress can also affect the brain's fear circuitry; however, pharmacological and non-pharmacological interventions can help reverse this damage (16,17).

The frontal EEG asymmetry-emotion model suggests that individuals with greater right frontal asymmetry exhibit higher anxiety symptoms (13). However, other researchers have found that the association between EEG asymmetry, withdrawal behavior and negative effects is more complex (18). Anxiety disorders are characterized by difficulties regulating emotional responses to perceived threats (19). This may be due to increased activity in the amygdala and other limbic/subcortical regions, decreased activity in the PFC and hippocampus, or a failure of top-down processes to regulate the ventral nervous system (20). Individuals with anxiety disorders often display a heightened sensitivity to threats or negative information in their environment, selectively attending to and experiencing increased amygdala activity in response to threats. There is evidence to indicate an association between anxiety disorders and heightened reactivity to threats and negative stimuli (21).

The hippocampus has provided valuable insight into understanding the impact of stress on the brain. Research has expanded to include interconnected regions, such as the amygdala and PFC (22). In rodents, chronic stress has been shown to lead to the degeneration of the PFC, specifically dendritic and spine loss in pyramidal cells, associated with impaired working memory (23). Notably, chronic stress has differential effects on different brain circuits. While dendritic growth increases in the amygdala, leading to an imbalance between the amygdala and prefrontal cortex function, the PFC neurons that form connections with other cortical areas undergo dendritic loss (23). However, neurons in the orbital prefrontal cortex and those that activate the amygdala do not atrophy during chronic stress. These findings highlight the complex effects of stress on brain structures and the interconnectedness of different brain regions (24).

A previous study discussed the association between reduced gray matter volume in the PFC and exposure to adverse events in humans. It also highlighted the impact of chronic stress on the functional connectivity and regulation of the PFC by the amygdala (23). Another study followed depressed adults over a period of 3 years and found that those who went into remission had less volume reduction in certain brain regions, such as the hippocampus and various regions of the PFC, compared to those who did not go into remission (25). The present study points out that internal and external factors were not fully controlled in the study and mentions that there was no control group for EEG data, which limits the certainty of attributing the changes observed to the treatment alone. The text emphasizes the need for further research to establish causality in the relationship between changes in EEG asymmetry and brain regions. It acknowledges that the data obtained from scales may suggest a relationship but recognizes these limitations.

In line with the results obtained from the present study, further studies need to be conducted in Turkey, to further examine the effectiveness of increasing levels of exposure and behavioral therapy for maladaptive behavior and anxiety. It is also recommended that this therapy be used in clinics as a psychotherapy method and presented as behavioral homework. It is also recommended that brain-based methods be added as an additional protocol to all psychotherapy methods, applied and recommended for other psychological problems other than anxiety. It is recommended that the findings in the present study should be confirmed by supported other studies and repeated in future studies with different samples or study groups.

## Figures and Tables

**Table I:** Descriptive analysis comparison of the sociodemographic characteristics of the participants in the experimental and control groups.

			Analyses		
Variable	Experimental group, n (%)	Control group, n (%)	χ² value	df	P-value
Sex					
Male	7 (46.7)	8 (53.3)	0.67	1	0.796
Female	8 (53.3)	7 (46.7)			
Total	15 (100.0)	15 (100.0)			
Age, years					
18-25	6 (40,0)	6 (40.0)	1.200	2	0.549
26-45	3 (20,0)	3 (20.0)			
≥46	6 (40,0)	6 (40.0)			
Total	15 (100.0)	15 (100.0)			
Level of education					
Primary school	6 (40.0)	6 (40.0)	2.300	3	0.506
Secondary school	3 (20.0)	3 (20.0)			
High school	2 (13.3)	2 (13.3)			
University	4 (26.7)	4 (26.7)			
Total	15 (100.0)	15 (100.0)	0.067	1	0.736
Drug use					
Yes	6 (40,0)	8 (53.3)			
No	9 (60,0)	7 (46.7)			
Total	15 (100.0)	15 (100.0)			
Marital status					
Single	7 (46.,7)	6 (40.0)	0.600	1	0.439
Married	8 (53.3)	9 (60.0)			
Total	15 (100.0)	15 (100.0)			

**Table II:** Results of repeated measures ANOVA of the pre-test, post-test and follow-up test scores of the study participants of the experimental group.

Source	Sum of squares	s.d	Mean squares	F value	P-value	Effect size	Difference
Intercept	56.711	28	2.025	29.122	0.001	0.530	2>1, 3>1 3=2
Measurement	67.489	2	33.744				
Error	64.889	56	1.159				
Total	189.089	86					

1, Pre-test; 2, post-test; 3, follow-up test.

**Table III:** Results of descriptive analysis of the pre-test, post-test and follow-up test scores of the experimental and control groups.

	Experimental group						Control group					
Dependent variable	Pre-test		Post-test		Follow-up		Pre-test		Post-test		Follow-up	
SSDKE	Mean	Sd	Mean	SS	Mean	Sd	Mean	SS	Mean	Sd	Mean	SS
	8.0667	1.53375	4.6667	1.58865	4.4667	1.84649	8.0667	0.45774	7.7333	0.25820	7.8667	0.35187

The Wilks' lambda value was 800 for the experimental group and 800 for the control group. The F value for the experimental group was 29.122. and the F value for the control was 1.625. P-values: 0.001 for the experimental group and 0.234 for the control group. SSDKE, Spielberger's State-Trait Anxiety Inventory.

**Table IV:** Results of ANCOVA for Spielberger's State-Trait Anxiety Inventory post-test scores according to sex.

Dependent variable: Post-test						
Source of variance	Sum of squares	df	Mean squares	F value	P-value	Effect size
Corrected model	10.057	3	3,352	1.459	0.279	0.285
Sex	1.936	1	1,936			
Pre-test	1.028	1	1,028			
re-test of sex	1.028	1	1,028			
Error	25.276	11	2,298			
Total	362.,000	15				
Corrected total	35.333	14				

The analysis was performed on the experimental groups. R^2^=0.285 (adjusted R^2^=0.090).

**Table V:** The changes in the right and left frontal and parietal lobe alpha frequencies (10-13 Hz) between the pre-test and post-test.

Group	10-13 Hz		Mean	n	Std. deviation	df	t	P-value
Experimental group	Frontal lobe	Pre-test right	2.0000	15	0.00000	14	1.344	0.05
		Post-test left	6.0000	15	0.81650			
	Parietal lobe	Pre-test right	2.5000	15	0.29099	14	3.111	0.05
		Post-test left	6.7500	15	1.50000			

## Data Availability

The datasets used and analyzed during the current study are available from the corresponding author upon reasonable request.
